# Primary genotoxicity in the liver following pulmonary exposure to carbon black nanoparticles in mice

**DOI:** 10.1186/s12989-017-0238-9

**Published:** 2018-01-03

**Authors:** Justyna Modrzynska, Trine Berthing, Gitte Ravn-Haren, Nicklas Raun Jacobsen, Ingrid Konow Weydahl, Katrin Loeschner, Alicja Mortensen, Anne Thoustrup Saber, Ulla Vogel

**Affiliations:** 1 Technical University of Denmark, National Food Institute, Lyngby, Denmark; 2 The National Research Centre for the Working Environment, Lersø Parkallé 105, DK-2100 Copenhagen Ø, Denmark; 3 Department of Micro- and Nanotechnology, Technical University of Denmark, Kongens Lyngby, Denmark

**Keywords:** Carbon black, Cerium oxide, Titanium dioxide, Nanoparticles, Liver, Intratracheal instillation, Intravenous injection, Oral gavage, Genotoxicity, DNA strand breaks

## Abstract

**Background:**

Little is known about the mechanism underlying the genotoxicity observed in the liver following pulmonary exposure to carbon black (CB) nanoparticles (NPs). The genotoxicity could be caused by the presence of translocated particles or by circulating inflammatory mediators released during pulmonary inflammation and acute-phase response. To address this, we evaluated induction of pulmonary inflammation, pulmonary and hepatic acute-phase response and genotoxicity following exposure to titanium dioxide (TiO_2_), cerium oxide (CeO_2_) or CB NPs. Female C57BL/6 mice were exposed by intratracheal instillation, intravenous injection or oral gavage to a single dose of 162 μg NPs/mouse and terminated 1, 28 or 180 days post-exposure alongside vehicle control.

**Results:**

Liver DNA damage assessed by the Comet Assay was observed after intravenous injection and intratracheal instillation of CB NPs but not after exposure to TiO_2_ or CeO_2_. Intratracheal exposure to NPs resulted in pulmonary inflammation in terms of increased neutrophils influx for all NPs 1 and 28 days post-exposure. Persistent pulmonary acute phase response was detected for all NPs at all three time points while only a transient induction of hepatic acute phase response was observed. All 3 materials were detected in the liver by enhanced darkfield microscopy up to 180 days post-exposure. In contrast to TiO_2_ and CeO_2_ NPs, CB NPs generated ROS in an acellular assay.

**Conclusions:**

Our results suggest that the observed hepatic DNA damage following intravenous and intratracheal dosing with CB NPs was caused by the presence of translocated, ROS-generating, particles detected in the liver rather than by the secondary effects of pulmonary inflammation or hepatic acute phase response.

**Electronic supplementary material:**

The online version of this article (10.1186/s12989-017-0238-9) contains supplementary material, which is available to authorized users.

## Background

The wide range of nanoparticles (NPs) applications leads to increased risk of unintended human exposure for consumers as well as for workers in the occupational environment. Inhaled NPs constitute a potential health risk [1] and therefore, understanding the fate and toxicity following pulmonary exposure to NPs has become an important issue.

Inhalation of particles leads to size dependent pulmonary deposition [2, 3]. A large fraction of the pulmonary deposited particles is removed from the upper airways by the mucociliary escalator and subsequently swallowed resulting in secondary exposure through the oral route [4–7]. In addition, a small fraction of the pulmonary deposited particles undergo translocation and primarily accumulate in the liver but also in other secondary organs [7–12].

Inhalation and intratracheal instillation of NPs induce pulmonary inflammation which is accompanied by a pulmonary acute phase response [13–15]. We have previously shown that instillation and inhalation of CB Printex 90 NPs induced hepatic genotoxicity in terms of increased DNA strand break levels and increased levels of oxidative DNA damage [14, 16–18]. However, the underlying mechanism is not clear. Exposure to particles can cause primary or secondary genotoxicity. Primary genotoxicity refers to DNA damage caused by direct physical interaction between particles and the genomic DNA and by ROS-mediated DNA damage in the absence of inflammation. Secondary genotoxicity refers to DNA damage as a result of action of reactive oxygen species (ROS) and reactive nitrogen species (RNS) as well as other secondary mediators (cytokines, chemokines) that are generated during particle-induced inflammation and acute phase response in the lungs that could initiate DNA damaging processes in the liver including induction of a hepatic acute phase response [19, 20].

The NPs used in this work, i.e. titanium dioxide (TiO_2_), cerium oxide (CeO_2_) and carbon black (CB) are all worldwide-used high-volume nanomaterials. Moreover, CB is a well-known ROS generator [21] and has been shown to be mutagenic [22]. TiO_2_-induced oxidative stress [23] and genotoxicity [24, 25] were also previously reported. In addition, CeO_2_-mediated DNA damages were confirmed in both in vivo [26] and in vitro [27] studies. CB NPs were included in the study as the DNA damaging test material in liver whereas TiO_2_ and CeO_2_ NPs were included as inflammogenic NPs with relatively low ROS generating abilities.

The objective of the present study was to assess the etiology of particle-induced genotoxicity in the liver. We therefore dosed mice with 3 different nanomaterials: TiO_2_, CeO_2_ and CB by pulmonary, oral and intravenous dosing to determine whether the observed hepatic DNA strand breaks are caused by secondary genotoxicity from pulmonary or hepatic inflammatory and acute phase responses or by direct genotoxicity from translocated NPs. Oral exposure was included to assess the contribution from the secondary oral exposure that accompanies particle clearance by the mucociliary escalator. Intravenous (IV) exposure was included to assess the effect of particle translocation and hepatic accumulation.

## Methods

### Animal study

Three hundred twenty four young adult C57BL/6 (B6JBOM-F) female mice were purchased from Taconic (Ry, Denmark) at 6 weeks of age and body weight of 17.5 ± 0.9 g (mean ± SD) and allowed to acclimate for 2 weeks before exposure. All animals were provided with the standard pellet diet (Altromin No. 1324) and acidified water ad libitum and housed in polypropylene cages with bedding and enrichment at controlled temperature 22 ± 1 °C and humidity of 55% ± 5 and with the reverse 12 h light/12 h dark cycle. The study was conducted in the agreement with the Danish Animal Experimental Inspectorate under the Ministry of Justice (Permission 2012–15–2934-00089 C6) and the Technical University of Denmark’s animal welfare protocol.

### Preparation of exposure stock

TiO_2_ was provided by NanoAmor, CeO_2_ was provided by Degussa-Quimidroga and CB (Printex 90) was provided by Evonik Degussa. Physicochemical characteristics of particles are presented in Table 1. All three materials were suspended in 2% *v*/v mouse serum from C57BL/6 mice in nanopure water to a final concentration of 3.24 mg/ml and dispersed by sonication for 15 min using Microson ultrasonic cell disruptor (XL-2000, Microson™, Qsonica, LLC.) equipped with disruptor horn with a diameter of 3.2 mm and maximum peak-to-peak amplitude of 180 μm. Suspensions were cooled on ice during the sonication procedure to prevent sample overheating. Control vehicle, 2% serum in nanopure water, was also sonicated prior to exposure as described above.

**Table 1:** Selected physicochemical parameters of the tested nanomaterials

	TiO_2_	CeO_2_	CB
Source	NanoAmor^b^	Degussa/Quimidroga^a^	Evonik-Degussa
Product form	Powder	Powder	Powder
Primary particle size	10.5 nm^b^	13.0 ± 12.1 nm^a^	14 nm^c^
Specific surface area	139.1 m^2^/g^b^	56.7 m^2^/g^a^	295 m^2^/g^d^
Particle density	4.23 g/cm^3 e^	7.29 g/cm^3 a^	2.1 g/cm^3 d^

^a^[62]; ^b^ [63];^c^ [29]; ^d^ [64]; ^e^ [65]

Dynamic light scattering (Malvern Zetasizer Nano ZS, Malvern Instruments, UK) was used to determine the hydrodynamic size distribution in the sonicated suspensions before administration. The suspensions were prepared as described above and measured in transparent 1 ml disposable cuvettes at the temperature of 25 °C. Six consecutively repeated measurements were performed. Duration of each measurement as well as attenuator index and measurement position within the cuvette were determined automatically by the instrument. For the calculation of the number-based particle size distribution, refractive index and extinction coefficient (absorption) for the tested materials were as followed: 2.9 and 0.1, respectively, for TiO_2_, 2.2 and 0.1, respectively, for CeO_2_, 2.02 and 2.0, respectively, for CB NPs.

### Exposure of mice

The mice (*n* = 9 per group) were given a single dose of 162 μg of TiO_2_, CeO_2_, or CB NPs suspension in a volume of 50 μl by intratracheal instillation, intravenous injection and oral gavage. Control mice received 50 μl of 2% serum (in nanopure water) that served as a vehicle for the preparation of NPs suspension. The dose used in the experiment was equal to the pulmonary deposition after nine 8 h working days at the Danish occupational exposure limit of 3.5 mg/m^3^ for CB [14]. Occupational exposure concentrations of up to 15 mg/m^3^ CB have been reported and the current dose would correspond to the pulmonary deposition during 2.2 work-days [28]. Mice subjected to intratracheal instillation and intravenous injection were anaesthetized with 0.5 ml/100 g body weight of Hypnorm® (Fentanyl citrate 0.315 mg/ml and Fluanisone 10 mg/ml, Nomeco) and Dormicum® (Midazolam 5 mg/ml, Roche) by subcutaneous injection in the neck prior the NP administration. Orally gavaged mice were not anaesthetized before the administration. Pulmonary exposed mice were dosed by intratracheal instillation as described previously [29]. In short, the sedated mice devoted for intratracheal instillation were placed on a 40° slope (upside down, with the head towards the floor) and a diode lamp was placed on the larynx to assure better visualization of the airways. The tongue was pressed down towards the lower jaw using a small spatula. 22 GA BD Insyte catheter (Becton Dickinson, Utah, USA) with a shortened needle used to intubate the trachea. The proper position of each catheter was confirmed by a highly sensitive pressure transducer. In a 250 μl SGE glass syringe (250F–LT-GT, MicroLab, Aarhus, Denmark) a volume of 50 μl of NP suspension followed by 200 μl air was instilled. Control groups received 50 μl of a control vehicle prepared as described above. After the removal of the catheter mice were placed back into vertical position with the head up to assure that the NP suspension remains in the lung and that the airways are unobstructed. We have previously shown the overall and even pulmonary distribution of particles using this exposure technique [30, 31]. Mice devoted for intravenous injection were restrained in plexiglas restraining tubes with the tail hanging out of the tube. Injection was performed using 0.4 x 20 mm needle (Terumo Europe, n.v. 3001, Leuven, Belgium). After the exposure, sedated mice were placed back to their cages, heated with a heating lamp and/or warming blanket and monitored until they fully recovered from anesthesia. Mice devoted for oral gavage were immobilized in a vertical position and the gavage needle was inserted into the esophagus and further toward the stomach to release the suspension of particles. After the exposure, mice returned to the cages and were closely monitored.

### Necropsy and cells & tissues collection

One, 28 and 180 days following administration of NPs mice were sedated by Hypnorm/Dormicum mixture (0.5–0.7 ml/100 g body weight) followed by the exsanguination by withdrawing the heart blood. Abdomen and thorax of mice were opened and macroscopic examination of all organs was performed. All observed abnormalities were noted. After withdrawal of the heart blood in the intratracheally instilled mice bronchoalveolar lavage (BAL) was performed (*N* = 6 mice/group). Lungs were flushed twice through the trachea with 0.8 ml of 0.9% sterile saline (NaCl) as described [32]. Each flush consisted of 3 up and down movements and was performed slowly (5–10 s each). The BAL was stored on ice until the centrifugation at 400 g for 10 min at 4 °C (Ole Dich centrifuge) was performed. The supernatant, BAL fluid (BALF), was collected in 1.5 ml tubes, snap-frozen in liquid nitrogen and stored at −80 °C until used. The BAL cells were re-suspended in 100 μl HAMS-F12 medium (HAMS-F12, GIBCO #21765, with 1% penicillin/streptomycin and 10% fetal bovine serum). BAL cells devoted for comet assay were prepared by the following procedure as described [32]: 40 μl of the cell suspension was mixed with 60 μl of cell culture freezing medium (HAMS-F12 with 1% penicillin/streptomycin, 10% fetal bovine serum and 10% of DMSO), divided into two 50 μl aliquots and stored at −80 °C until used. The total number of cells presented in the BAL fluid was determined using the Nucleo-Counter NC-100 with NucleoCassette™ (Chemometec, Allerød, Denmark). 20 μl of the cell suspension was mixed with 180 μl HAMS F12 medium, suspension was divided into 2 aliquots and live/dead cells were counted according to the manufacturer’s instructions. For the estimation of cellular composition in BAL fluid rest of the cell suspension (approximately 40 μl) was deposited on the microscope slide by centrifugation at room temperature for 10 min. at 550 g in a Cytofuge® 2 (StatSpin, TRIOLAB, Brønby, Denmark). The slides were fixed in 96% ethanol for 5 min and stained with May-Grünwald-Giemsa dye using standard staining protocol. The cellular composition was quantified on 200 cells under light microscope (100 x magnifications). The liver (*N* = 9/group) and lungs (from BAL flushed mice, *N* = 6/group) were divided into specific pieces, snap frozen in liquid nitrogen in cryotubes (NUNC) and stored at −80 °C for later use. For all mice throughout the whole experiment samples were taken from the same parts of organs. Specimens from livers (N = 9/group) and lungs (from mice that had not been used for BALF collection, *N* = 3/group) were fixed in 4% neutral buffered formaldehyde, paraffin-embedded and 4 μm thick sections were stained with hematoxylin and eosin for microscopical examination.

### Brightfield and darkfield microscopy

Cytoviva enhanced darkfield hyperspectral system (Auburn, AL, USA) was used to detect particles in the liver tissue, by scanning histological sections at 40 x in enhanced darkfield mode. Darkfield images were acquired at 40 x and brightfield images were acquired at 40× and 100 x on an Olympus BX 43 microscope with a Qimaging Retiga4000R camera. Uneven illumination in brightfield images was corrected using ImageJ [33] and the Calculator Plus plugin via the formula: Corrected_image = (image / background) * 255. The background image was a maximum projection of 3 background brightfield images without tissue.

### Preparation of mRNA and cDNA from the liver and lung tissue

Total RNA was isolated from the frozen liver and lung tissue (16–20 mg) on Maxwell ® 16 (Promega, USA) using Maxwell® 16 LEV simply RNA Tissue Kit (AS1280, Promega, USA) following the manufacturer’s protocol. Tissue lysis was performed by vigorously shaking the samples using a Tissuelyser (Qiagen, Denmark) with a 5 mm stainless steel beads for 60 s. DEPC-treated nuclease-free water was used to elute the mRNA. Complementary DNA (cDNA) was prepared using TaqMan® reverse transcription reagents (Applied Biosystem, USA) as stated in manufacturer’s protocol. Concentration of total mRNA was determined by NanoDrop 2000c (ThermoFisher, USA).

### Real-time RT-PCR

Gene expression levels of *Saa1* in the liver tissue and *Saa3* in the lung tissue were assessed using quantitative PCR as described previously [25, 34]. 18S RNA served as a reference gene. Each of the analyzed samples was run in triplicates using ViiA7 Real-Time PCR detector (Applied Biosystem, USA). TaqMan® predeveloped reagents were used throughout the analysis. Target and reference gene expression was quantified in triplicates in separate wells. Target gene expression was determined by the comparative method 2^- ΔCt^.

### Comet assay

DNA strand break levels were quantified by the comet assay in the liver and lung tissue and BAL cells as described in [35]. In brief, single-cell suspension of the liver and lung tissue was obtained by the homogenization of the deep frozen liver and lung piece in an ice-cold Merchant’s buffer through a stainless steel mesh (diameter 0.5 cm, mesh size 0.4 mm) mounted on a syringe, to yield individual cells. The BAL fluid cells were thawed in a 37 °C water bath before diluting with the Mechant’s buffer without the filtration step. The cell suspension was embedded in a low melting point agarose (0.7%) and deposited on microscope Travigen 20-Well CometSlides™. The slides were immersed in a lysing solution and stored overnight at 4 °C. Subsequently, samples were treated with an alkaline buffer and alkaline electrophoresis with circulating ice-cold electrophoresis buffer was performed (25 min, 38 V/cm, 0.700 A). Thereafter, slides were neutralized in neutralization buffer (0.4 M Tris, pH 7.5), fixed with 96% ethanol and stained with a fluorescent DNA intercalating dye SYBRGreen®. DNA strand breaks determined as the % of DNA in the comet tail (%TDNA) and as the comet tail length (TL) were scored by the fully automated PatchFinder™ system (IMSTAR, France). In order to control the day-to-day variation and to ensure equal electrophoresis efficiency both negative (A549 human lung epithelial cell line treated with PBS for 30 min at 4 °C) and positive (A549 human lung epithelial cell line treated with 60 μM H_2_O_2_ for 30 min at 4 °C,) controls were included. The high throughput comet assay analysis allowed analysis of all related sample on the same day decreasing potential variation due to the different electrophoresis or the duration of the incubation procedure.

### Determination of ROS-generating ability of NPs

Assessment of NPs ability to generate ROS was performed in vitro in a cell-free environment as described previously [21, 36]. Briefly, 500 μl of 1 mM 2′,7′-dichlorodihydrofluorescein diacetate (DCFH_2_-DA) (Invitrogen) was chemically hydrolyzed with 2 ml of 0.01 M NaOH for 30 min to 2′,7′-dichlorodihydrofluorescein (DCFH_2_). DCFH_2_-DA probe is light sensitive therefore the experiment was conducted in a dark environment. The formed DCFH_2_ was further diluted with 10 ml of 25 mM phosphate buffer (pH 7.4) to 0.04 mM. The NPs’ ability to generate ROS production was determined in Hank’s balanced saline solution (HBSS, without phenol) using a final DCFH_2_ concentration of 0.01 mM. Prior to the assay, NPs were sonicated (Branson S-450D) for 16 min without a pause and further diluted in HBSS. Generated ROS caused formation of 2′,7′-dichlorofluorescein (DCF) from DCFH_2_ that was spectrofluorimetrically measured following 3 h of incubation in the dark (37 °C and 5% CO_2_). Excitation and emission wavelengths were λ_ex_ = 490 nm and λ_em_ = 520 nm, respectively (Victor Wallac-2 1420; PerkinElmer, Skovlunde, Denmark).

### Statistical analysis

All presented values are expressed as mean ± standard deviation (SD) unless differently stated. One-way or two-way analysis of variance (ANOVA) was used to analyze the data sets. In order to fulfil the normality and variance homogeneity criteria some variables were logarithmically transformed. Non-normally distributed data were ranked before applying nonparametric one-way or two-way ANOVA analysis. If the statistical significance was reached in the ANOVA analysis, Tukey *post-hoc* multiple comparison test was used to test the differences between the test groups. *P*-value ≤0.05 was considered significant. All statistical analyses were calculated using SAS 9.4 statistical software (SAS Institute Inc., Cary, NC, USA).

## Results

### DLS

**Fig. 1:**
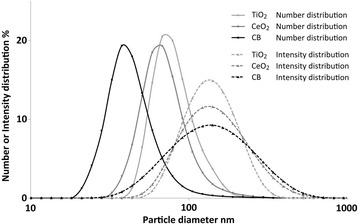
Dynamic light scattering intensity size and number-based size distributions of TiO_2_, CeO_2_ and CB NPs in suspension (3.24 mg/ml in 2% serum in nanopure water)

Dynamic light scattering (DLS) was used to determine particle agglomerate size of TiO_2_, CeO_2_ and CB NPs suspensions. The both the hydrodynamic number-based size distribution revealed a narrow, unimodal peak with the average diameter below 100 nm for all particle suspensions (Fig. 1). The intensity size distributions also showed unimodal peaks (Fig. 1). The median particle diameter measured for TiO_2_, CeO_2_ and CB NPs was 68 nm, 68 nm, and 51 nm, respectively. Z-average and polydispersity index were 131.4 and 0.120, respectively, for TiO_2_, 148.8 and 0.174, respectively, for CeO_2_, 104.3 and 0.157, respectively, for CB NPs.

### BAL fluid cellular composition

**Fig. 2:**
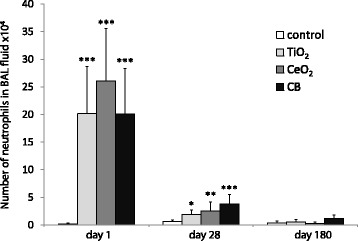
Number of neutrophils detected in BAL fluid following intratracheal instillation of 162 μg of TiO_2_, CeO_2_ or CB NPs measured 1, 28 and 180 days after exposure. Particle exposed groups *n* = 6, vehicle control *n* = 6. All values are presented as mean + SD. An asterisk (*) denotes *P* ≤ 0.05, (**) *P* ≤ 0.01, (***) *P* ≤ 0.001 of neutrophil levels in exposed groups versus vehicle control

Analysis of BAL fluid cellular composition following pulmonary exposure to the NPs revealed that intratracheal instillation of all three particles resulted in increased numbers of total cell counts after day 1 as well as increased neutrophil influx in lungs 1 and 28 days post-exposure (Fig. 2 and Table 2). Elevation in number of total cell counts after day 1 as well as increased number of neutrophils at day 1 and day 28 is consistent with previous studies [29, 37, 38]. The distributions of total cells, neutrophils, macrophages, lymphocytes, eosinophils are shown in Table 2.

**Table 2:** BAL fluid cellular composition following intratracheal instillation of 162 μg of TiO_2_, CeO_2_ or CB NPs 1, 28 and 180 days post-exposure

BAL fluid cellular composition after day 1				
	Control	TiO_2_	CeO_2_	CB
Total cells (× 10^5^)	0.9 ± 0.6	2.5 ± 0.9**	3.4 ± 1.1***	2.4 ± 1.0**
Neutrophils (× 10^4^)	0.2 ± 0.2	20.2 ± 8.5***	26.1 ± 9.5***	20.1 ± 8.3***
Macrophages (× 10^4^)	9.0 ± 5.7	4.5 ± 1.9	6.1 ± 1.5	2.9 ± 1.5**
Lymphocytes (× 10^2^)	1.9 ± 2.9	0.0 ± 0.0	7.1 ± 7.9	0.0 ± 0.0
Eosinophils (×10^3^)	0.0 ± 0.1	3.0 ± 2.5**	13.3 ± 9.2***	7.6 ± 6.8**
BAL fluid cellular composition after day 28				
	Control	TiO_2_	CeO_2_	CB
Total cells (× 10^5^)	1.4 ± 0.5	1.1 ± 0.2	1.6 ± 0.7	1.6 ± 0.8
Neutrophils (×10^4^)	0.6 ± 0.3	1.9 ± 0.9*	2.6 ± 1.6**	3.8 ± 1.7***
Macrophages (×10^4^)	7.9 ± 2.0	9.3 ± 2.3	12.9 ± 5.5	11.9 ± 5.5
Lymphocytes (×10^2^)	8.5 ± 13.7	35.5 ± 44.3	28.3 ± 55.4	53.7 ± 78.1
Eosinophils (×10^3^)	51.2 ± 39.3	0.1 ± 0.2**	0.2 ± 0.3*	0.0 ± 0.0***
BAL fluid cellular composition after day 180				
	Control	TiO_2_	CeO_2_	CB
Total cells (× 10^5^)	0.7 ± 0.3	1.0 ± 0.2	0.9 ± 0.3	1.3 ± 0.6
Neutrophils (×10^4^)	0.3 ± 0.4	0.6 ± 0.4	0.3 ± 0.3	1.1 ± 0.7
Macrophages (×10^4^)	7.0 ± 2.9	9.4 ± 2.1	8.4 ± 2.9	11.9 ± 4.9
Lymphocytes (×10^2^)	0.0 ± 0.0	0.0 ± 0.0	0.5 ± 1.1	0.0 ± 0.0
Eosinophils (×10^3^)	0.3 ± 0.4	0.4 ± 0.5	4.9 ± 6.8	4.9 ± 4.4

Particle exposed groups *n* = 6, vehicle control *n* = 6. All values are presented as mean ± SD. An asterisk (*) denotes *P* ≤ 0.05, (**) *P* ≤ 0.01, (***) *P* ≤ 0.001 of statistically significantly different cells level in exposed groups versus vehicle control

### *Saa3* mRNA expression in the lung tissue

**Fig. 3:**
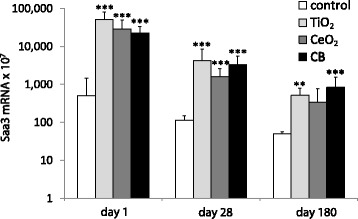
Pulmonary *Saa3* mRNA expression levels in mice following intratracheal instillation of 162 μg of TiO_2_, CeO_2_ or CB NPs measured 1, 28 and 180 days after the exposure. *Saa3* mRNA levels were normalized to 18S rRNA. Particle exposed groups *n* = 6, vehicle control *n* = 6. All values are presented as mean + SD. Asterisks (**) denote *P* ≤ 0.01, (***) P ≤ 0.001 of *Saa3* mRNA level in exposed groups versus vehicle control

Pulmonary acute phase response was assessed as *Saa3* mRNA expression levels [39, 40]. Analysis of *Saa3* gene expression revealed acute phase response in the lungs following intratracheal instillation of TiO_2_, CeO_2_ and CB NPs at all three time points. After day 1 and day 28, pulmonary *Saa3* mRNA levels were statistically significantly increased compared to the vehicle control for all three particles (*P* ≤ 0.001) with no differences between particles. After day 180, pulmonary *Saa3* mRNA levels were statistically significantly increased for TiO_2_ (*P* ≤ 0.01) and CB (*P* ≤ 0.001) groups with no observed differences between particles (Fig. 3 and Additional file 1).

Overall, our results indicate that pulmonary exposure to all three NPs induces pulmonary inflammation and pulmonary acute phase response.

### Hepatic presence of NPs

**Fig. 4:**
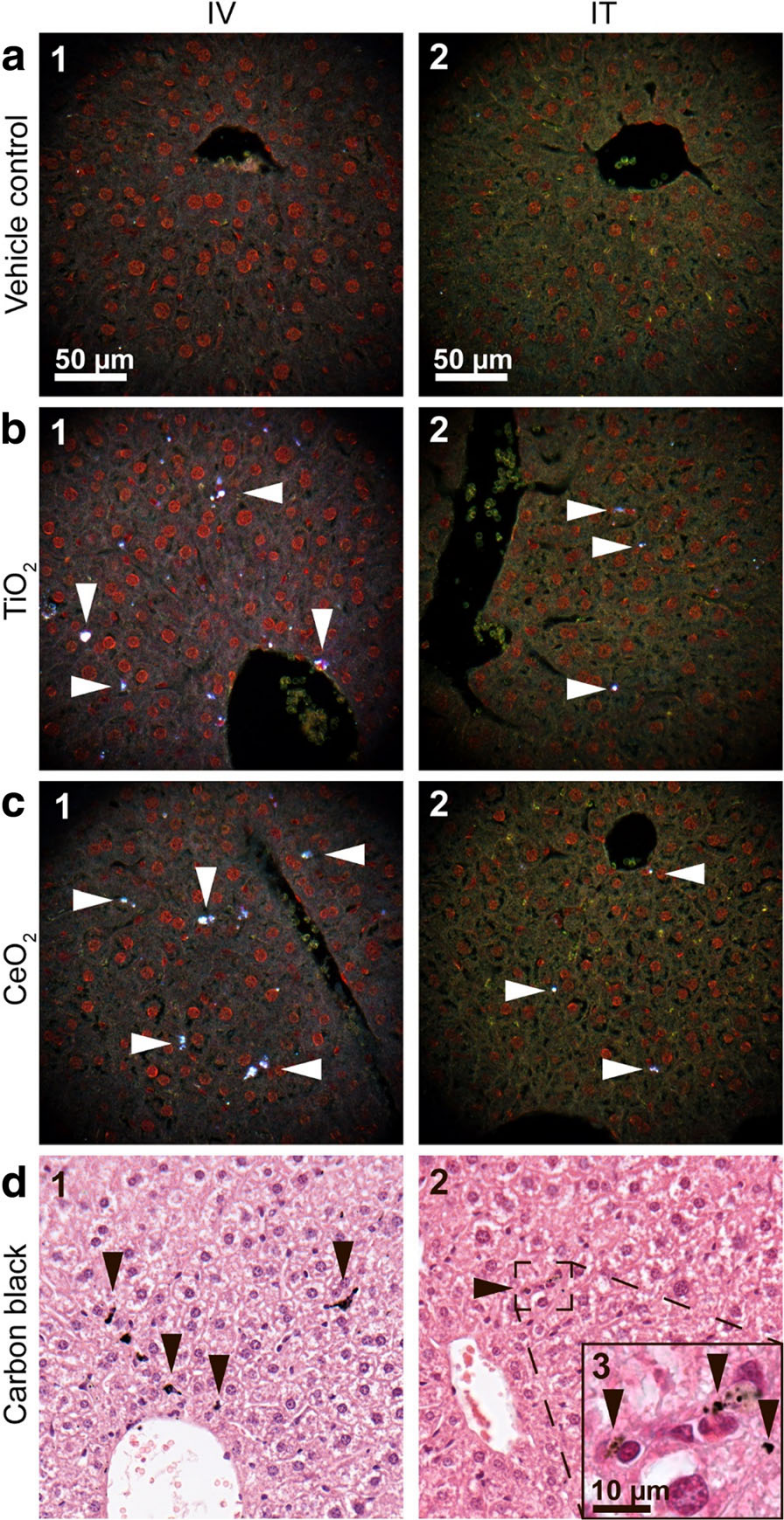
Enhanced darkfield (**a**-**c**) and brightfield (**d**) pictures of H&E stained liver tissue from intravenously (IV) and intratracheally (IT) exposed mice that received control vehicle (**a**) or 162 μg/animal of TiO_2_ (**b**), CeO_2_ (**c**) or CB (**d**) NPs 180 days post-exposure. In the liver sections of mice exposed to TiO_2_ and CeO_2_ light scattering aggregates were observed using enhanced darkfield microscopy (**b** and **c**, selected NPs marked with white arrowheads). In the liver sections of mice exposed to CB NPs black aggregates were observed using brightfield microscopy (**d**, selected NPs marked with black arrowheads)

Hepatic detection of NPs 180 days after intravenous injection and intratracheal instillation by enhanced darkfield microscopy and brightfield microscopy is presented in Fig. 4. Aggregates of foreign material, visible as white spots were present in liver tissue from mice exposed to TiO_2_ and CeO_2_ NPs (Fig. 4B and C, selected NPs marked with white arrowheads). In the livers from mice exposed to CB NPs, black aggregates of foreign material were present (Fig. 4D, selected NPs marked with black arrowheads). Following IT exposure (Fig. 4B-D, panel 2) foreign material aggregates were smaller and much less frequent than aggregates detected in the liver tissue after IV exposure (Fig. B-D, panel 1). In all exposure groups aggregates were primarily detected in sinusoids and partly perivascular and often appeared to be phagocytized by Kupffer cells. Black or white spots were occasionally also seen in vehicle controls, but the appearance and location indicated artefacts from tissue preparation. No exposure related foreign material aggregates were identified following oral exposure (results not shown.)

### *Saa1* mRNA expression in liver tissue

**Fig. 5:**
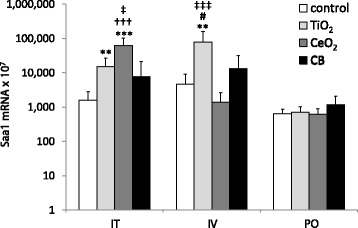
Hepatic *Saa1* mRNA expression levels in mice following intratracheal instillation (I.T.), intravenous injection (I.V.) and oral gavage (P.O.) of 162 μg of TiO_2_, CeO_2_ or CB measured 1 day after exposure. Particle exposed groups *n* = 9, vehicle control *n* = 9. All values are presented as mean + SD. Asterisks (**) denote *P* ≤ 0.01 and (***) *P* ≤ 0.001 of *Saa1* mRNA levels in exposed groups versus vehicle control. Hashtag (#) denotes *P* ≤ 0.05 of *Saa1* mRNA level in TiO_2_ vs CB groups. Crosses (†††) denote P ≤ 0.001 of *Saa1* mRNA level in CeO_2_ vs CB groups. Double cross (‡) denotes *P* ≤ 0.05 and (‡‡‡) denote *P* ≤ 0.001 of *Saa1* mRNA level in TiO_2_ vs CeO_2_ groups

Hepatic acute phase response was assessed by the measurement of *Saa1* mRNA expression levels [39]. Analysis of *Saa1* gene expression revealed hepatic acute phase response on day 1 following intratracheal instillation and intravenous injection (Fig. 5 and Additional file 2). No NP-related effects on hepatic *Saa1* expression levels were observed at later time points.

One day following intratracheal instillation, hepatic *Saa1* mRNA levels were statistically significantly increased for TiO_2_ (*P* ≤ 0.01) and CeO_2_ (*P* ≤ 0.001) exposed groups as compared to the vehicle control. CeO_2_ NPs exposure induced statistically significant higher hepatic *Saa1* mRNA levels than TiO_2_ (*P* ≤ 0.05) and CB (*P* ≤ 0.001).

One day following intravenous injection, hepatic *Saa1* mRNA level was statistically significantly increased for TiO_2_ exposed mice compared to the vehicle control (*P* ≤ 0.01). TiO_2_ exposure induced statistically significantly higher hepatic *Saa1* mRNA levels compared to CeO_2_ (P ≤ 0.001) and CB (*P* ≤ 0.05) exposure.

### DNA strand breaks in liver and lung

**Fig. 6:**
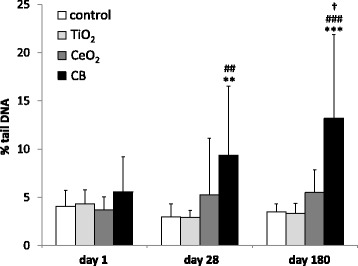
DNA strand break levels in the liver tissue (% tail DNA) detected by comet assay following intratracheal instillation of 162 μg of TiO_2_, CeO_2_, CB measured 1, 28 and 180 days after exposure. Particle exposed groups *n* = 9, vehicle control *n* = 9. All values are presented as mean + SD. Asterisks (**) denote *P* ≤ 0.01, (***) denote *P* ≤ 0.001 of DNA SB level in exposed groups versus vehicle control. Hashtags (##) denote *P* ≤ 0.01, (###) denote *P* ≤ 0.001 of DNA SB level in TiO_2_ vs CB groups. Cross (†) denotes *P* ≤ 0.05 of DNA SB level in CeO_2_ vs CB groups

**Fig. 7:**
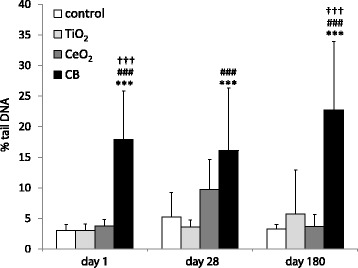
DNA strand break levels in the liver tissue (% tail DNA) detected by comet assay following intravenous injection of 162 μg of TiO_2_, CeO_2_ or CB NPs measured 1, 28 and 180 days after exposure. Particle exposed groups *n* = 9, vehicle control *n* = 9. All values are presented as mean + SD. Asterisks (***) denote *P* ≤ 0.001 of DNA SB level in exposed groups versus vehicle control. Hashtags (###) denote *P* ≤ 0.001 of DNA SB level in TiO_2_ vs CB groups. Crosses (†††) denote *P* ≤ 0.001 of DNA SB level in CeO_2_ vs CB groups

DNA strand break levels were determined in the liver and lung tissue as well as in the BAL cells using comet assay (Tables 3 and 4 and Figs. 6 and 7).

**Table 3:** DNA strand break levels (% tail DNA) in liver tissue following intratracheal instillation, intravenous injection and oral gavage of 162 μg of TiO_2_, CeO_2_ or CB NPs 1, 28 and 180 days after the exposure determined by the Comet assay

DNA SB in the liver following intratracheal instillation				
	Control	TiO_2_	CeO_2_	CB
day 1	4.1 ± 4.6	4.3 ± 1.5	3.7 ± 1.4	5.6 ± 3.7
day 28	3.0 ± 1.4	2.9 ± 0.8	5.2 ± 5.9	9.4 ± 7.2**^##^
day 180	3.5 ± 0.8	3.4 ± 1.0	5.5 ± 2.4	12.2 ± 8.7***^###†^
DNA SB in the liver following intravenous injection				
	Control	TiO_2_	CeO_2_	CB
day 1	3.1 ± 0.9	3.0 ± 1.0	3.7 ± 1.1	17.9 ± 7.9***^###†††^
day 28	5.2 ± 4.0	3.6 ± 1.1	9.7 ± 4.9	16.1 ± 10.2***^###^
day 180	3.3 ± 0.7	5.8 ± 7.2	3.7 ± 2.0	22.8 ± 11.1***^###†††^
DNA SB in the liver following oral gavage				
	Control	TiO_2_	CeO_2_	CB
day 1	5.5 ± 3.8	4.6 ± 3.5	7.9 ± 8.6	13.9 ± 10.4
day 28	3.0 ± 0.6	2.8 ± 0.6	3.3 ± 0.7	2.4 ± 0.6
day 180	2.8 ± 0.8	3.5 ± 0.9	3.8 ± 3.5	7.2 ± 6.5

Particle exposed groups *n* = 9, vehicle control *n* = 9. All values are presented as mean ± SD. Asterisks (**) denote *P* ≤ 0.01, (***) *P* ≤ 0.001 of DNA SB levels in exposed groups versus vehicle control. Hashtags (##) denote *P* ≤ 0.01 and (###) *P* ≤ 0.001 of DNA SB levels in the CB groups compared to TiO_2_ groups. Cross (†) denotes *P* ≤ 0.05, (†††) *P* ≤ 0.001 of DNA SB levels in the CB groups compared to CeO_2_ groups

**Table 4:** DNA strand break levels (% tail DNA) in lung tissue 1, 28 and 180 days following intratracheal instillation and from the BAL cells 1 and 28 days following intratracheal instillation of 162 μg of TiO_2_, CeO_2_ or CB determined by the Comet assay

DNA SB in the lungs following intratracheal instillation				
	Control	TiO_2_	CeO_2_	CB
day 1	3.3 ± 0.8	3.2 ± 0.9	3.8 ± 0.6	4.6 ± 1.0
day 28	6.1 ± 1.3	5.9 ± 0.9	5.5 ± 1.1	5.3 ± 1.2
day 180	3.6 ± 1.0	5.2 ± 0.9*	4.2 ± 1.1	5.1 ± 1.4
DNA SB in the BAL cells following intratracheal instillation				
	Control	TiO_2_	CeO_2_	CB
day 1	4.5 ± 0.7	4.4 ± 0.9	5.6 ± 1.3	5.8 ± 1.2
day 28	5.9 ± 1.1	4.0 ± 1.0	4.3 ± 1.0	4.0 ± 1.4

Particle exposed groups *n* = 6, vehicle control *n* = 6. All values are presented as mean ± SD. An asterisk (*) denotes *P* ≤ 0.05 of DNA SB levels in exposed groups versus vehicle control

In the pulmonary exposed groups DNA strand break levels in the liver tissue expressed as % tail DNA (%TDNA) were statistically significantly increased compared to the vehicle control in the CB group (3.2 fold increase, *P* ≤ 0.01) 28 days following exposure (Fig. 6). CB exposure also induced statistically significantly higher DNA strand break levels than TiO_2_ exposure (3.2 fold, *P* ≤ 0.01). 180 days after intratracheal instillation %TDNA was statistically significantly increased for CB exposed group compared to vehicle control (3.8 fold, *P* ≤ 0.001), TiO_2_ (3.9 fold, *P* ≤ 0.001) and CeO_2_ (2.4 fold, *P* ≤ 0.05) (Fig. 6 and Table 3).

Analysis of the %TDNA in liver tissue in intravenously exposed groups revealed statistically significantly differences between CB and vehicle control and between CB and TiO_2_ and CeO_2_ exposed groups (5.8, 5.9 and 4.8 fold increase, respectively, *P* ≤ 0.0001 for all groups) 1 day following intravenous injection (Fig. 7). 28 days post-exposure, DNA strand break levels in liver tissue in the CB group were statistically significantly increased compared to vehicle control (3.1 fold, *P* ≤ 0.001) and TiO_2_ exposed group (4.4 fold, P ≤ 0.0001). 180 days following exposure, DNA strand break levels in liver tissue in the CB group were statistically significantly increased compared to vehicle control and TiO_2_ and CeO_2_ exposed groups (6.9, 4.0 and 6.2 fold, respectively, *P* ≤ 0.0001 for all groups) (Table 3 and Fig. 7).

In the orally exposed groups %TDNA levels the liver tissue were unaffected by exposure at all assessed time points (Table 3).

In the lung tissue after intratracheal instillation %TDNA was unaffected by the exposure after day 1 and day 28 whereas significant increase in %TDNA was measured in TiO_2_ group compared to vehicle control (1.5 fold increase, *P* ≤ 0.05) 180 days post instillation (Table 4). In the BAL cells %TDNA was unaffected by the exposure after day 1 and day 28 (Table 4). DNA strand breaks in the BAL cells 180 days following exposure were not analyzed as the samples were lost.

### ROS-generation assay

**Fig. 8:**
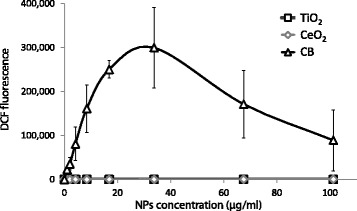
NPs-induced generation of reactive oxygen species (ROS) measured in a cell-free assay. All values are presented as mean +/− SD from 3 independent measurements

Among analyzed NPs, CB exhibited the greatest ability to generate ROS (Fig. 8). CeO_2_ NPs also generated ROS but the level was negligible and only marginally elevated above background. There was no signal detected for TiO_2_ at any of the analyzed concentrations.

## Discussion

We have previously observed that pulmonary exposure to reactive NPs induced genotoxicity in the liver tissue [14, 17, 25, 41]. Therefore, in the present work, we wanted to determine whether the observed hepatic DNA strand breaks following pulmonary exposure to CB NPs were caused by primary genotoxicity due to the direct effects of translocated particles and their ability to induce reactive oxygen species formation [21] or by secondary genotoxicity caused by pulmonary inflammation and acute phase response triggering molecular signaling cascade (release of cytokines, chemokines and ROS/RNS) that, in turn, initiate downstream toxic effects in the liver [41, 42].

### Hepatic genotoxicity and possible mechanism for the adverse hepatic effects

We found evidence of particle translocation from lung to liver for all three NPs using darkfield and brigthfield microscopy. In relation to the current study, we have also quantified the extrapulmonary translocation of CeO_2_ and TiO_2_ NPs following intratracheal exposure using inductively coupled plasma mass spectrometry (ICP-MS) and confirmed translocation of CeO_2_ and TiO_2_ to the liver (Modrzynska et al., submitted for publication).

In the present study, intravenous injection of CB NPs resulted in increased DNA strand break (SB) levels in liver 1, 28 and 180 days post-exposure, whereas intratracheal instillation of CB induced DNA SBs in liver 28 and 180 days post-exposure. Significant translocation of TiO_2_ NPs from lung to liver was recently demonstrated using radioactively labelled TiO_2_ NPs [7]. In agreement with this, we also detected intratracheally instilled TiO_2_ and CeO_2_ NPs in the liver 180 days post-exposure by darkfield microscopy. The fact that hepatic genotoxicity was only observed at the later time points following pulmonary exposure might reflect the time needed for particles to translocate in sufficient amounts from lungs to the liver [7].

It has been previously suggested by Jackson [41] that the observed liver DNA SBs could be a result of gastrointestinal tract exposure due to the whole-body inhalation and further fur grooming. Minute uptake of TiO_2_ NPs following oral exposure has been reported [7, 43, 44] and it was recently estimated that up to 20% of TiO_2_ NPs accumulated in liver following pulmonary exposure had reached the liver by the oral route [7]. However, we have found no detectable translocation of CeO_2_ and TiO_2_ NPs in the liver after oral exposure. Overall, no hepatotoxic effects were observed following oral gavage since the dosing did not elicit neither acute phase response nor genotoxicity in the liver. This indicates that the secondary oral exposure caused by swallowing a portion of lung-deposited particles did not contribute to the observed adverse effects. In general, larger dose or repeated oral exposure were needed to evoke the toxic effect observed in the other studies [26, 45, 46].

CB is a well characterized carbonaceous nanomaterial and very potent ROS generator [22, 47, 48]. Exposure to CB NPs has been shown to induce oxidative DNA damage, increased levels of DNA strand breaks [21, 49] as well as mutagenicity in vivo and in vitro [22, 50]. The mutation spectrum observed in vitro is consistent with being caused by oxidative DNA damage [47]. The aforementioned studies are in a good agreement with the present results, showing that CB is much more potent in terms of generating ROS than TiO_2_ and CeO_2_ NPs. Moreover, CB NPs contain very low levels of organic impurities [22, 51, 52] that excludes the hypothesis that CB-induced genotoxicity is caused by polycyclic aromatic hydrocarbons (PAH) that are absorbed on particles’ surface.

### Pulmonary acute phase response but no hepatic acute phase response

In our previous studies [13, 15, 40, 53, 54] we demonstrated that *Saa3* was the most upregulated acute phase gene during pulmonary acute phase response and, frequently, the most differentially expressed gene. *Saa1* was the most differentially expressed acute phase response gene in the liver [39]. Therefore, *Saa3* mRNA expression levels were used as a biomarker for pulmonary acute phase response whereas *Saa1* expression was used as a biomarker for hepatic acute phase response [55].

Pulmonary exposure to all three NPs induced similar pulmonary inflammation in terms of neutrophil influx that persisted up to day 28 and long-lasting pulmonary acute phase response that sustained for 180 days. The highest induction of acute phase was observed after day 1 and declined in a time-dependent manner remaining elevated for TiO_2_ and CB NPs 180 days following exposure. Pulmonary acute phase response was similar for all three particles when compared to the control and no differences between particles were observed. Thus, if the hepatic genotoxicity was caused by systemic circulation of pulmonary inflammatory messengers, then all the assessed NPs would induce hepatic genotoxicity. Instead, hepatic genotoxicity was only observed in CB exposed groups. Therefore, it is unlikely that circulating cytokines released by pulmonary-mediated inflammation and acute phase response caused the DNA lesions. Moreover, a recent meta-analysis of global transcriptional pattern following pulmonary exposure to CB and TiO_2_ NPs indicated that exposure to CB and TiO_2_ induced similar changes in pulmonary transcription [56]. Both materials evoked comparable neutrophilic-dominated lung inflammation and similarly altered expression level of many pro-inflammatory genes. In addition, our previous studies indicated that CB [16] and TiO_2_ [53] NPs induced similar alterations in the expression profile of selected cytokines genes in the lung tissue. Demonstrated resemblances in the elicited pulmonary response following exposure to CB and TiO_2_ should lead to the similar induction level of different secondary mediators that would cause similar level of hepatic DNA SBs in both exposure groups. Instead, we have only observed hepatic genotoxicity in the CB NPs exposed mice, which is an additional evidence of primary genotoxicity caused by the deposited CB NPs rather than by the effects of circulating secondary mediators.

In the present study, we found induction of hepatic acute phase response following lung instillation of TiO_2_ NPs and CeO_2_ NPs and following intravenous injection of TiO_2_ NPs that was present at day 1 but has already subsided by the day 28. Induction of hepatic acute phase response was much weaker and shorter than the pulmonary acute-phase response. Pulmonary exposure to TiO_2_ increased pulmonary *Saa3* expression level 100- fold whereas 17.3 fold induction and 9.4 fold induction of hepatic *Saa1* was measured after intravenous injection and intratracheal instillation, respectively. No acute phase response was detected in the liver of orally gavaged animals. In addition CB NPs exposure by IV or IT, in contrast to TiO_2_ and CeO_2_ did not induce hepatic acute phase response, therefore hepatic acute phase response is also not a likely cause of the observed genotoxicity.

We have previously reported transient or no hepatic acute phase response following pulmonary exposure measured by analysis of the transcriptional changes in liver mRNA of mice exposed to diesel exhaust particles (DEP), CB, multi walled carbon nanotubes [13, 39]. Transient acute phase response in the liver tissue was also reported after pulmonary exposure to 162 μg/mouse of TiO_2_ as reported by [25]. Hepatic acute phase response induced by intravenous injection of gold particles has been reported by Zhang and co-workers [57]. They used *Saa* reporter mouse model to detect transcriptional activation of hepatic *Saa* using bioluminescence imaging. Their results demonstrated that among all of the assessed particles 50 nm Au nanospheres exhibited the highest capacity to induce activation of hepatic *Saa*. The highest activation was detected 4 h following intravenous injection. The signal was transient and gradually declined. In the present study lack of detection of hepatic acute phase response after intravenous injection of CeO_2_ and CB at all three time points as well as temporary induction of acute phase response in the TiO_2_ group on day 1 could be explained by the brief and transient nature of acute phase response induction in the liver that causes difficulties in the measurement.

Overall, our results suggest that the observed hepatic DNA damage following IV and IT exposure to CB was caused by the presence of translocated NPs in the liver and their ability to induce oxidative stress rather than by secondary effects of pulmonary inflammation and acute phase response. Particles deposited in the liver are primary accumulated in the Kupffer cells [58]. In the current study, we observed foreign material in sinusoids and in Kupffer cells. Only a small fraction of the liver cells appeared to be directly exposed to the particle-generated ROS [41]. Apparently, this was sufficient to induce hepatic genotoxicity.

Our results may also imply that inhaled carbon nanoparticles could induce hepatic genotoxicity leading to liver carcinogenesis. It is supported by epidemiological studies showing that exposure to traffic related air pollution is associated with risk of liver cancer [59, 60]. The International Agency for Research on Cancer (IARC) has classified CB NPs as possibly carcinogenic to humans [61]. It can be speculated that the presence of CB NPs in the liver may, in a long-time perspective, contribute to the onset of cancer.

## Conclusions

Pulmonary exposure to three different NPs, TiO_2_, CeO_2_ and CB induced long lasting pulmonary inflammation and acute phase response. Indications of translocation to the liver were found for all three NPs. However, only CB dosed by IT and IV induced hepatic genotoxicity. Therefore, our findings indicate that hepatic DNA strand breaks following pulmonary exposure to CB are likely caused by direct effects of CB NPs that translocate from lung to liver rather than being caused by inflammatory or acute phase responses. Furthermore, the lack of particle translocation, inflammation and acute phase response following oral exposure to NPs suggest that the secondary oral exposure following pulmonary clearance of inhaled particles does not contribute to the observed hepatic genotoxicity.

## Additional files

Additional file 1: Pulmonary *Saa3* mRNA expression level following intratracheal instillation of 162 μg of TiO_2_, CeO_2_ or CB NPs 1, 28 and 180 days post-exposure. (DOCX 14 kb)

Additional file 2: Hepatic *Saa1* mRNA expression level following intratracheal instillation, intravenous injection and oral gavage of 162 μg of TiO_2_, CeO_2_ or CB NPs 1, 28 and 180 days post-exposure. (DOCX 15 kb)

## Abbreviations

%TDNA: % of the DNA in the comet tail
BAL: Bronchoalveolar lavage
BFM: Brightfield microscopy
CB: Carbon clack
CeO_2_: Cerium oxide
DLS: Dynamic light scattering
DNA SBs: DNA strand breaks
EDFM: Enhanced darkfield microscopy
ICP-MS: Inductively coupled plasma mass spectrometry
IL-1: Interleukin 1
IL-6: Interleukin 6
IT: Intratracheal
IV: Intravenous
NPs: Nanoparticles
PO: Per oral
RNS: Reactive nitrogen species
ROS: Reactive oxygen species
RT-PCR: Reverse transcription polymerase chain reaction
SAA: Serum amyloid A
TiO_2_: Titanium dioxide
TL: Tail length
TNFα: Tumor necrosis factor α
