# Supplementary material for: Primary genotoxicity in the liver following pulmonary exposure to carbon black nanoparticles in mice
Source: Part Fibre Toxicol. 2018 Jan 3;15:2. doi: 10.1186/s12989-017-0238-9 (PMC5753473; doi:10.1186/s12989-017-0238-9)
Supplement: Supplementary file 1 — Pulmonary Saa3 mRNA expression level following intratracheal instillation of 162 μg of TiO2, CeO2 or CB NPs 1, 28 and 180 days post-exposure. (DOCX 14 kb) [file 12989_2017_238_MOESM1_ESM.docx]

**Additional file 1.** Pulmonary *Saa3* mRNA expression level following intratracheal instillation of 162 µg of TiO_2_, CeO_2_ or CB NPs 1, 28 and 180 days post-exposure

| **Pulmonary *Saa3* mRNA expression following intratracheal instillation** | | | | |
| --- | --- | --- | --- | --- |
|  | Control | TiO_2_ | CeO_2_ | CB |
| day 1 | 511 ± 938 | 51977 ± 28702*** | 28509 ± 20645*** | 22295 ± 11663*** |
| day 28 | 112 ± 36 | 4227 ± 4388*** | 1623 ± 1003*** | 3297 ± 2204*** |
| day 180 | 51 ± 6 | 515 ± 288** | 344 ± 433 | 838 ± 715*** |

Particle exposed groups n=6, vehicle control n=6. All values are presented as mean ± SD. Asterisks (**) denote P ≤ 0.01, (***) P ≤ 0.001 of *Saa3* mRNA level in exposed groups versus vehicle control.
