# Supplementary material for: Primary genotoxicity in the liver following pulmonary exposure to carbon black nanoparticles in mice
Source: Part Fibre Toxicol. 2018 Jan 3;15:2. doi: 10.1186/s12989-017-0238-9 (PMC5753473; doi:10.1186/s12989-017-0238-9)
Supplement: Supplementary file 2 — Hepatic Saa1 mRNA expression level following intratracheal instillation, intravenous injection and oral gavage of 162 μg of TiO2, CeO2 or CB NPs 1, 28 and 180 days post-exposure. (DOCX 15 kb) [file 12989_2017_238_MOESM2_ESM.docx]

**Additional file 2.** Hepatic *Saa1* mRNA expression level following intratracheal instillation, intravenous injection and oral gavage of 162 µg of TiO_2_, CeO_2_ or CB NPs 1, 28 and 180 days post-exposure

| **Hepatic *Saa1* mRNA expression following intratracheal instillation** | | | | |
| --- | --- | --- | --- | --- |
|  | Control | TiO_2_ | CeO_2_ | CB |
| day 1 | 1608 ± 1250 | ** 15044 ± 12053 | ***†††‡ 62975 ± 40137 | 7649 ± 13853 |
| day 28 | 1388 ± 634 | 1321 ± 512 | 852 ± 724 | 943 ± 590 |
| day 180 | 671 ± 242 | 1311 ± 1122 | 852 ± 556 | 682 ± 147 |
| **Hepatic *Saa1* mRNA expression following intravenous injection** | | | | |
|  | Control | TiO_2_ | CeO_2_ | CB |
| day 1 | 4601 ± 4707 | **#‡‡‡ 79551 ± 80452 | 1410 ± 1213 | 13115 ± 19003 |
| day 28 | 961 ± 524 | 753 ± 419 | 542 ± 255 | 543 ± 306 |
| day 180 | 592 ± 387 | 732 ± 739 | 910 ± 445 | 848 ± 351 |
| **Hepatic *Saa1* mRNA expression following oral gavage** | | | | |
|  | Control | TiO_2_ | CeO_2_ | CB |
| day 1 | 649 ± 228 | 715 ± 306 | 625 ± 260 | 1158 ± 923 |
| day 28 | 875 ± 485 | 650 ± 315 | 654 ± 249 | 332 ± 125 |
| day 180 | 945 ± 1322 | 751 ± 553 | 984 ± 1324 | 677 ± 576 |

Particle exposed groups n=9, vehicle control n=9. Messenger RNA expression levels were normalized to 18S rRNA. All values are presented as mean ± SD. Asterisks (**) denote P ≤ 0.01 and (***) P ≤ 0.001 of *Saa1* mRNA level in exposed groups versus vehicle control. Hashtag (#) denotes P ≤ 0.05 of *Saa1* mRNA level in TiO_2_ vs CB groups. Crosses (†††) denote P ≤ 0.001 and below of *Saa1* mRNA level in CeO_2_ vs CB groups. Double cross (‡) denotes P ≤ 0.05 and (‡‡‡) denote P ≤ 0.001 of *Saa1* mRNA level in TiO_2_ vs CeO_2_ groups.
